# The impact of China’s home and community-based older adult care reform policies on the mental health of older adults: a nationwide quasi-experimental study

**DOI:** 10.3389/fpubh.2026.1764174

**Published:** 2026-04-10

**Authors:** Xuefei Xia, Feilong Wang, Shang Xie, Jishu Xian, Xiaoying Chen

**Affiliations:** 1 Department of Neurosurgery, The First Affiliated Hospital of Army Medical University (Southwest Hospital), Chongqing, China; 2 School of Business, Hubei University, Wuhan, China

**Keywords:** China, depressive symptoms, home- and community-based older adult care, mental health, older adults

## Abstract

**Objective:**

The study aims to examine the effects of pilot reform policies related to home- and community-based care services on the mental health of older adults in China. This research carries significant practical implications for achieving the goal of “aging well” and unleashing the potential of the “silver economy.”

**Methods:**

Using data from the China Family Panel Studies (CFPS) spanning 2010–2022, this study treats the pilot reform of home- and community-based older adult care services as a quasi-experiment and applies the difference-in-differences (DID) method for causal identification. It quantitatively evaluates the impact of integrated home- and community-based care on the mental health of older adults.

**Results:**

The findings indicate that: (1) The pilot reform of home- and community-based older adult care services significantly reduced depressive symptoms and improved mental health among older adults. (2) Better physical health and enhanced perception of filial support represent key mechanisms through which the pilot policy exerts positive effects on older adults’ mental health. (3) Heterogeneity analysis shows that the policy effects are particularly pronounced among females, individuals with lower education levels, and those with disabilities.

**Conclusion:**

The reform of home- and community-based older adult care services has the potential to significantly improve the mental health of older adults. These findings are not only important for the understanding of the integration of home-based and community-based older adult care services, but also provide valuable insights for the development of older adult care service systems in developing countries.

## 1 Introduction

The global population is aging at an accelerated pace, posing significant challenges for China. By the end of 2024, the number of individuals aged 60 and older in China had reached 310 million, accounting for 22.0% of the total population. This group is characterized by a large base and a rapid growth rate (1). Older adults often experience physical decline, reduced social engagement, and adverse life events such as bereavement, making them particularly vulnerable to mental health issues, including anxiety, depression, and loneliness (2). The incidence of moderate-to-severe anxiety among community-dwelling older adults has been reported to be 56.3% (341 out of 606), and the prevalence of moderate-to-severe depression stands at 28.7% (174 out of 606) (3). Approximately 90% of older adults in China rely on traditional home-based care as their primary source of support (4), where intergenerational family interactions play a crucial role in promoting their mental health (5). However, rapid population aging, coupled with declining fertility rates, has weakened China’s traditional family support system. Consequently, it has become increasingly difficult to meet the growing demand for older adult care services through familial care alone. As a result, a growing number of older adults—particularly those with disabilities (6) or those living alone (7)—face challenges such as inadequate daily care and a lack of emotional support, which can negatively impact their mental health.

In response to declining birth rates, population aging, and shrinking family sizes, China has implemented home- and community-based older adult care (HCEC) policies. These policies enable older adults to age in place within a familiar environment, maintain their social networks, and preserve family connections. This approach not only alleviates feelings of helplessness and the risk of solitary death among empty-nest older adults but also mitigates the loneliness and emotional detachment often associated with institutional care. Owing to its alignment with Chinese cultural values, public preferences, cost-effectiveness, and potential to reduce health inequality, HCEC has emerged as a promising framework for older adult care (8).

**Figure 1:**
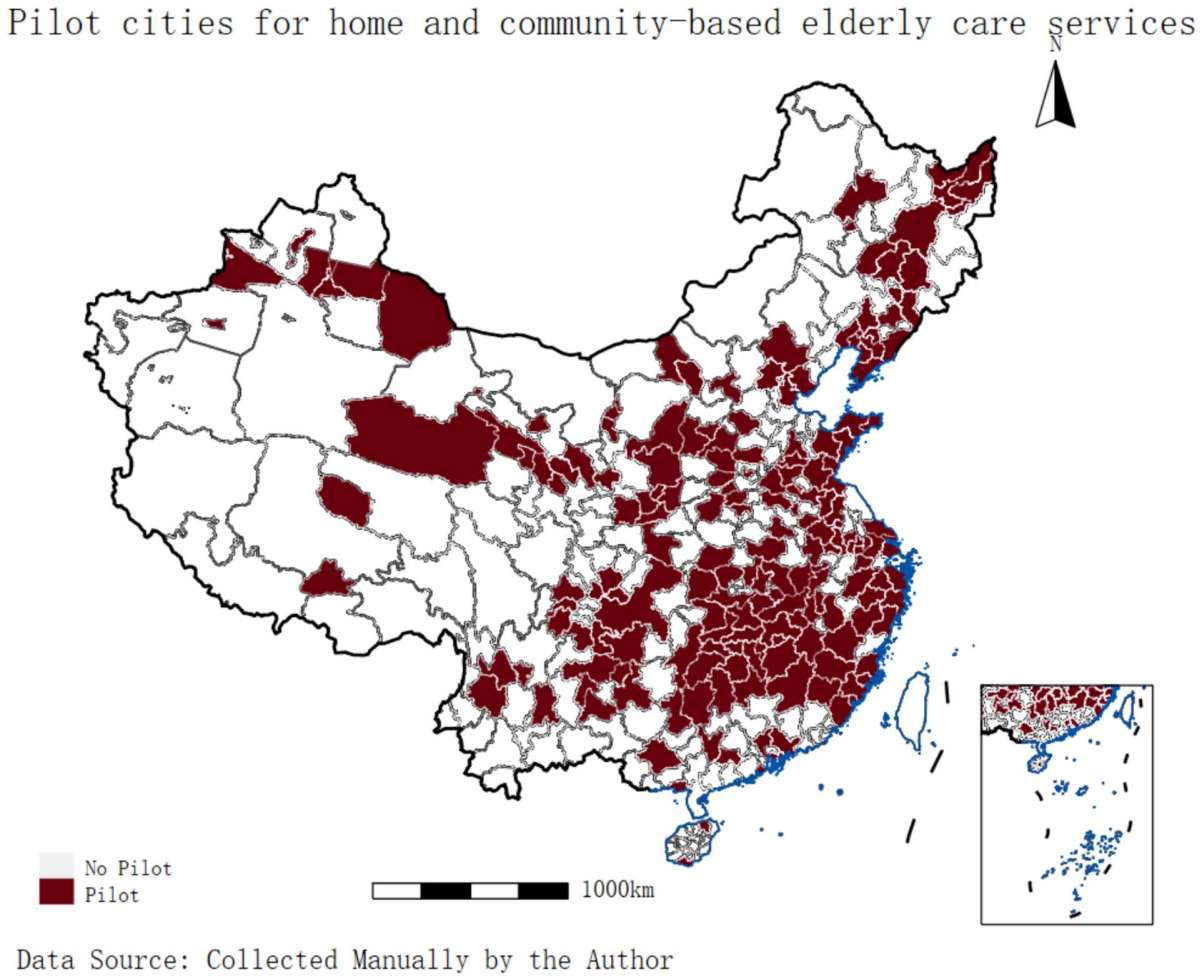
Distribution map of pilot cities for HCEC. The first batch (end of 2016) included 26 pilot cities. The second batch (2017) included 28 pilot cities. The third batch (2019) included 35 pilot cities. The fourth batch (2020–2023) included 54 pilot cities. The fifth batch (2020) included 50 pilot cities. The white blocks indicate cities that were not included in the pilot programme.

Numerous studies have highlighted the strong demand for HCEC services among older adults, particularly for daily assistance and emotional support (9). Furthermore, the care needs of older adults are individualized and shaped by various factors, including education level, health status, family environment, economic conditions, and social security (10). Existing studies evaluating policy effects have mainly focused on health status, quality of life, and life satisfaction among older adults (11). Improving the mental health of older adults is a key goal in advancing healthy aging. To further safeguard the physical and mental well-being of older adults and enhance their quality of life, China’s Ministry of Civil Affairs and Ministry of Finance issued the Notice on Central Financial Support for Pilot Reforms of Home- and Community-Based Elderly Care Services in 2016. From 2016 to 2020, the central government allocated 5 billion yuan to support five batches of pilot regions, covering 203 cities (districts) in 31 provinces, which accounted for 60% of all prefecture-level cities in China (Figure 1).

Despite these developments, research investigating the impact of HCEC pilot policies on the mental health of older adults remains limited, especially with regard to their medium- and long-term effects. Clarifying the effects of these policies is consistent with the principles of active aging and healthy aging, and also addresses older adults’ care preferences and the concept of “aging in community.”This study is of great practical importance for promoting the high-quality development of older adult care. Accordingly, this study aims to examine the effects of HCEC service reform pilots on the mental health of older adults, focusing on an in-depth exploration of the underlying mechanisms and medium- to long-term policy effects.

## 2 Methods

### 2.1 Data sources and processing

This study utilized data from the China Family Panel Studies (CFPS) database covering the period 2010–2022, which coincides with the implementation period of all five batches of pilot policies. This design allows for a comprehensive and scientific investigation of the medium- to long-term dynamic impacts of HCEC on the mental health of older adults in China. In addition, we manually collected data on home- and community-based older adult care pilot policies, including the lists of prefecture-level cities (including municipalities) covered in the first to fifth batches of pilot reforms and their corresponding implementation timelines. These data were then matched with the CFPS data for empirical analysis.

The CFPS data were processed and filtered according to the following procedures:

Only individuals aged 60 and above were included in the analysis. Household-level information was matched using unique individual identification codes.

Geographic Exclusion: Observations without valid district or county information were excluded.

Policy Timing Alignment: Given that the first batch of pilots was announced in November 2016, 2016 CFPS observations with ambiguous policy exposure status were excluded to ensure the validity of the pretreatment parallel trend assumption.

Longitudinal Robustness: Only individuals with three or more waves of observations were retained.

After the above screening, the final sample included 9,444 older adults and 37,141 person-year observations, covering 125 cities (in this study, municipalities and counties are also treated as cities).

### 2.2 Variable definitions

#### 2.2.1 Dependent variable: mental health level

The depression scores of respondents were used to reflect the mental health status of older adults, with lower depression scores indicating better mental health. To ensure comparability across different waves of data collection, raw depression scores were standardized to Z-scores (mean = 0, standard deviation = 1).

#### 2.2.2 Core explanatory variable: pilot program for home- and community-based older adult care service reform

The core explanatory variable is a binary indicator that denotes whether an individual was affected by the home- and community-based older adult care service reform pilot. If respondents lived in designated pilot cities and were interviewed after the policy was issued, the difference-in-differences (DID) variable was assigned a value of 1 (treatment group); otherwise, it was assigned a value of 0 (control group). This definition of the policy variable (DID) facilitated the evaluation of the implementation effects of the home- and community-based older adult care service reform. Panel B of Table 1 presents descriptive statistics for the treatment group and the pilot policy variables. From 2010 to 2022, 66 cities and municipalities in the sample were included in the home- and community-based older adult care service reform pilots, with approximately 16% of the surveyed older adults affected by this policy.

**Table 1:** Descriptive statistics of participants.

Variables	Total participants		Treatment group		Control group	
	Panel A Characteristics of demographic					
Gender(1 = Male, 0 = Female)	0.49	0.50	0.48	0.50	0.50	0.50
Age (years, Mean, SD)	69.70	7.07	69.90	7.18	69.50	6.96
Education level (Years)	3.79	4.50	4.13	4.57	3.44	4.40
Marital status (1 = married, 0 = not married)	0.71	0.45	0.72	0.45	0.70	0.46
Family members (Mean, SD)	3.95	2.13	3.78	2.05	4.11	2.20
Number of children (Mean, SD)	0.54	0.62	0.51	0.61	0.56	0.63
Annual household income (RMB/year)	15784.83	40372.17	16807.47	30634.99	14761.52	48163.96
Household consumption(RMB/Yuan)	55130.18	90687.55	56971.83	79843.29	53290.30	100326.26
House property rights (=1 yes, 0 = no)	0.87	0.34	0.85	0.35	0.88	0.33
Residential properties (Mean, SD)	1.06	0.59	1.06	0.61	1.07	0.58
Non-clean energy usage (1 = yes, 0 = no)	0.41	0.49	0.35	0.48	0.46	0.50
Clean water usage (1 = yes, 0 = no)	0.69	0.46	0.72	0.45	0.66	0.47
Panel B Core variables and independent variables						
Standardized depression score (Mean, SD)	0.00	1.00	−0.02	0.98	0.02	1.02
Treatment group (1 = yes, 0 = no)	0.50	0.50	1.00	0.00	0.00	0.00
DID(1 = yes, 0 = no)	0.16	0.36	0.32	0.47	0.00	0.00
Panel C Main mechanism variables						
Self-rated health status (1–7: very unhealthy–very healthy)	2.54	1.28	2.59	1.26	2.50	1.30
Illness (1 = yes, 0 = no)	0.40	0.49	0.40	0.49	0.41	0.49
Disease Severity (0–3: not sick - very sick)	0.92	1.22	0.90	1.21	0.95	1.23
Hospitalization within 1 year (1 = yes, 0 = no)	0.20	0.40	0.21	0.40	0.19	0.39
Frequencies of visit between children and parents (Mean, SD)	7.41	6.59	7.50	6.52	7.33	6.67
Frequencies of communication between children and parents (Mean, SD)	7.23	6.60	7.22	6.51	7.23	6.69

Family-level data come from the CFPS family economic database. DID, difference-in-differences.

#### 2.2.3 Controlled variables

To mitigate omitted-variable bias, control variables were included at two levels.Individual Level: Gender, Age, Years of Education, Marital Status.

Household Level: Household size, number of co-residing children, annual household income per capita, total household consumption, homeownership status (binary), number of properties owned, non-clean energy usage (binary), and access to clean water (binary). Descriptive statistics are summarized in Table 1 (Panel A).

#### 2.2.4 Mechanism variables

We constructed the mechanism analysis indicators of this paper from two pathways based on the CFPS database.

Physical Health: Self-rated health (ordinal), occurrence of illness (binary), hospitalization status (binary), severity of illness (scale).

Filial Support: Frequency of In-Person Meetings and Communication with Children (Continuous). The measures are detailed in Table 1 (Panel C).

### 2.3 Identification strategy

Based on relevant policy documents from the Ministry of Civil Affairs and the Ministry of Finance, we compiled a list of pilot cities for home- and community-based older adult care service reform since 2016. Each city, including municipalities, that has been officially designated as a pilot area for home- and community-based older adult care service reform served as the starting point for policy implementation. We defined the areas on the pilot city list as the treatment group and the non-pilot cities as the control group. The research subjects consisted of older adults in both pilot and non-pilot areas, and we employed the difference-in-differences (DID) method to assess the impact of the pilot on the mental health of older adults. Given that the pilot reforms for home- and community-based older adult care services were announced in multiple batches and at various time points, a multi-period DID approach was developed to accurately identify the policy’s impact on the mental health of older adults. Additionally, a possible issue is that the selection of pilot cities for the home- and community-based older adult care program is not random. Instead, the choice of pilot cities depends on factors such as local economic development, the degree of population aging, and labor market structure. For instance, cities experiencing faster economic growth usually have greater fiscal capacity to support the rollout of pilot policies. To mitigate this self-selection bias, this study adopted the identification approach suggested by Gruber (12). Specifically, we included a set of city-level variables measured before the pilot period, interacted with linear time trends, in the regression model. The four pre-pilot city-level variables considered as potentially influencing pilot selection were: gross regional product (GRP, in 100 million RMB), the proportion of older residents, the percentage of the population holding non-agricultural hukou, and the employment rate among people aged 16 and older. Collectively, these variables effectively captured regional differences in economic status, population aging, and employment patterns. The baseline regression model was specified as follows:


$$\text{Scor}e_{i,j,t}=\beta_{0}+\beta_{1}\mathrm{DI}D_{i,j,t}+\sum \beta_{2}X_{i,j,t}+\gamma_{j}+\lambda_{t}+u_{\mathrm{jt}}+\varepsilon_{i,j,t}$$ (1)

-
$\text{Scor}e_{i,j,t}$ : depression score of individual *i* in city *j* at year *t*.
-
$\mathrm{DI}D_{i,j,t}$ : Binary policy indicator (If the individual lives in the pilot area, the value is 1; otherwise, it is 0).
-
$X_{i,j,t}$ : Vector of individual and household controls.
-
$Y_{j}$ : City fixed effects (alleviate the potential estimation bias caused by the population migration of some individuals between cities).
-
$\lambda_{t}$ : Year fixed effects (controls for the possible impact of significant policies and events in certain years).
-
$u_{\mathrm{jt}}$ : Time trends of city-level characteristic variables.
-
$\varepsilon_{i,j,t}$ : the regression error terms (employs robust standard errors clustered at the city - year level, to address potential heteroscedasticity and autocorrelation issues).

The coefficient β1 captures the average treatment effect on mental health of older adults.

This study also addressed the non-randomness in the selection of national policy pilot cities. A balance test at the individual level was performed to examine whether significant differences existed between the control and treatment groups prior to policy implementation. Using data from 2016 (the year immediately preceding policy enactment) as the baseline, we assessed the intergroup differences in the core dependent variable and individual/household characteristics that may affect policy outcomes (see Table 2). Columns (1)–(2) report the mean and standard deviation of the control group in the baseline year, Columns (3)–(4) show those of the treatment group, and Columns (5)–(6) display the intergroup differences between the two groups.

**Table 2:** Balance test.

Variables	Controlled group		Treatment group		Difference	
	Mean	Standard deviation	Mean	Standard deviation	Mean	Standard error
	(1)	(2)	(3)	(4)	(5)	(6)
	Differences in individual-level characteristics in 2016					
Mental health	0.057	1.012	−0.054	0.986	−0.111*	0.003
Gender(1 = male,0 = female)	0.491	0.500	0.481	0.500	−0.01	0.000
Age	69.363	7.012	69.909	7.359	0.546**	0.001
Education level	3.335	4.368	3.925	4.511	0.591*	0.004
Marital status	0.755	0.430	0.768	0.422	0.014	0.000
Household size	4.154	2.228	3.806	2.030	−0.348**	0.007
Number of children	0.577	0.637	0.540	0.621	−0.038	0.001
Household per capita net income (yuan/year)	12905.418	28788.431	14580.368	15661.200	1674.95	0.001
Household consumption level	59904.357	92841.651	66089.200	86269.892	6184.84	0.001
Home ownership(1 yes 0 no)	0.886	0.318	0.857	0.350	−0.029*	0.002
Number of residential properties	1.095	0.580	1.064	0.598	−0.03	0.001
Non-clean energy usage	0.489	0.500	0.332	0.471	−0.158**	0.026
Clean water usage	0.686	0.464	0.724	0.447	0.037	0.002

* indicate statistically significant results: *p* < 0.1; ** indicate statistically significant results: *p* < 0.05; *** indicate statistically significant results: *p* < 0.01.

Specifically, the core dependent variable exhibited significant intergroup differences, indicating potential disparities in mental health among older adults between pilot and non-pilot cities. Most control variables related to individual characteristics showed no significant intergroup differences, suggesting that non-random selection bias at the individual level was negligible. However, significant differences were observed in age, household size, and household non-clean energy use: the treatment group comprised older individuals, smaller household sizes, and a lower proportion of non-clean energy consumption. To mitigate regression bias arising from omitted individual and household characteristics, we controlled for the 12 individual/household variables in subsequent analyses. In addition, time trends of pre-pilot city-level characteristics were controlled to ensure the credibility and robustness of the empirical findings.

## 3 Results

### 3.1 Baseline regression results

This study utilized the Difference-in-Differences (DID) model to assess the impact of the HCEC on the mental health of older adults, using data from the China Family Panel Studies (CFPS) database. The baseline regression results, presented in Table 3, were derived from Equation 1. Column (1) includes year fixed effects and individual/household demographic characteristics. Column (2) further incorporates time trends of city-level characteristic variables. In addition, Column (3) adds city fixed effects to verify the robustness of the regression results, and Column (3) serves as the baseline regression for this study. The findings consistently indicate that the pilot policies significantly reduce depression scores, with Column (3) showing a decrease of 0.100 standard units (*p* < 0.01).

**Table 3:** The impact of home and community care reform on the mental health of the older.

Variables	Standardized depression score			
	(1)	(2)	(3)	(4)
*DID*	−0.099***	−0.095***	−0.100***	−0.073**
	(0.030)	(0.030)	(0.034)	(0.036)
Individual and household variables	Controlled	Controlled	Controlled	Controlled
City-level characteristic time trends		Controlled	Controlled	Controlled
Fixed effects (year)	Controlled	Controlled	Controlled	Controlled
Fixed effects (city)			Controlled	Controlled
City-level characteristic variable				Controlled
*f*	0.021	0.022	0.038	0.046
Sample size (number)	34,375	34,375	34,375	32,450

(1) ** indicate statistically significant results: *p* < 0.05; *** indicate statistically significant results: *p* < 0.01. (2) All data in columns are the results of cluster regression at the district or county level. (3) DID: difference-in-differences. (4) Unless further annotation, the following controlled variables are the same as in Table 3.

Furthermore, although this study incorporates time trends of pre-pilot city characteristics in the baseline regression to mitigate the issue of non-random selection of pilot cities, city-level characteristics at the time of the survey—such as economic development, fiscal capacity, and aging rate—may also affect the implementation effect of the HCEC policy. Therefore, following common practices in the literature, this study further includes five variables to control for the influence of macroeconomic and social factors: city GDP (100 million yuan), the share of the secondary industry in GDP, the share of the tertiary industry in GDP, local general public budget revenue, and the urban aging rate. The results are reported in Column (4). The estimates in Column (4) show that the effect of the HCEC policy remains statistically significant after controlling for various urban socioeconomic variables, which further validates the robustness of the main conclusions.

Given the extensive missing values for city-level characteristic variables in many ethnic minority areas, the reduction in sample size may affect the accuracy of the policy evaluation. Thus, the regression specification in Column (3) is adopted as the baseline regression in this paper.

### 3.2 Parallel trend test

The foundational hypothesis of the baseline Equation 1 posits that, in the absence of the pilot policy’s influence, the trends in mental health levels for both the treatment group and the control group of older adults should exhibit parallelism. To evaluate this parallel trend, we employed a non-parametric event study methodology, incorporating an interaction term that combined each year dummy variable with the treatment group into the baseline Equation 1. This approach facilitated dynamic effect analysis and an ex-ante parallel trend assessment through event study methodology. Furthermore, in alignment with established methodologies in the literature (11), the study designated the year preceding the implementation of the pilot policy as the benchmark group for analysis, thereby enabling the observation of changes in the coefficients of each interaction term. The specific regression setup for the parallel trend test is shown in Equation (2):


$$\begin{array}{l} \text{Scor}e_{i,j,t}=\alpha_{0}+\sum_{k\neq -1}\alpha_{1}\text{Trea}t_{i,j}\times \mathrm{Wav}e_{t_{0}+k}+\\ \sum \alpha_{2}X_{i,j,t}+\gamma_{j}+\lambda_{t}+u_{\mathrm{jt}}+\varepsilon_{i,j,t} \end{array}$$ (2)

The analysis involved a binary variable that was categorized based on the geographical region of the individual at the time the pilot policy was implemented. This variable $\text{Trea}t_{i,j}$ indicates whether city *j*, where individual *i* resides, is designated as a policy area for the home- and community-based older adult care service reform pilot, with values of 1 or 0. The variable $\mathrm{Wav}e_{t_{0}+k}$ is the time indicator, where the initial year t0 of the pilot policy, and represents the k-th year following the implementation of the pilot policy. This study examined five periods preceding the implementation of the pilot policy, the current period, and two subsequent periods, specifically within the range of −5 ≤ *k* ≤ 2. The coefficient $\alpha_{1}$ corresponds to the policy interaction term, and the dynamic effect of the pilot policy was assessed by analyzing the temporal changes in $\alpha_{1}$ , the other parameters were designated consistently with Equation 1.

**Figure 2:**
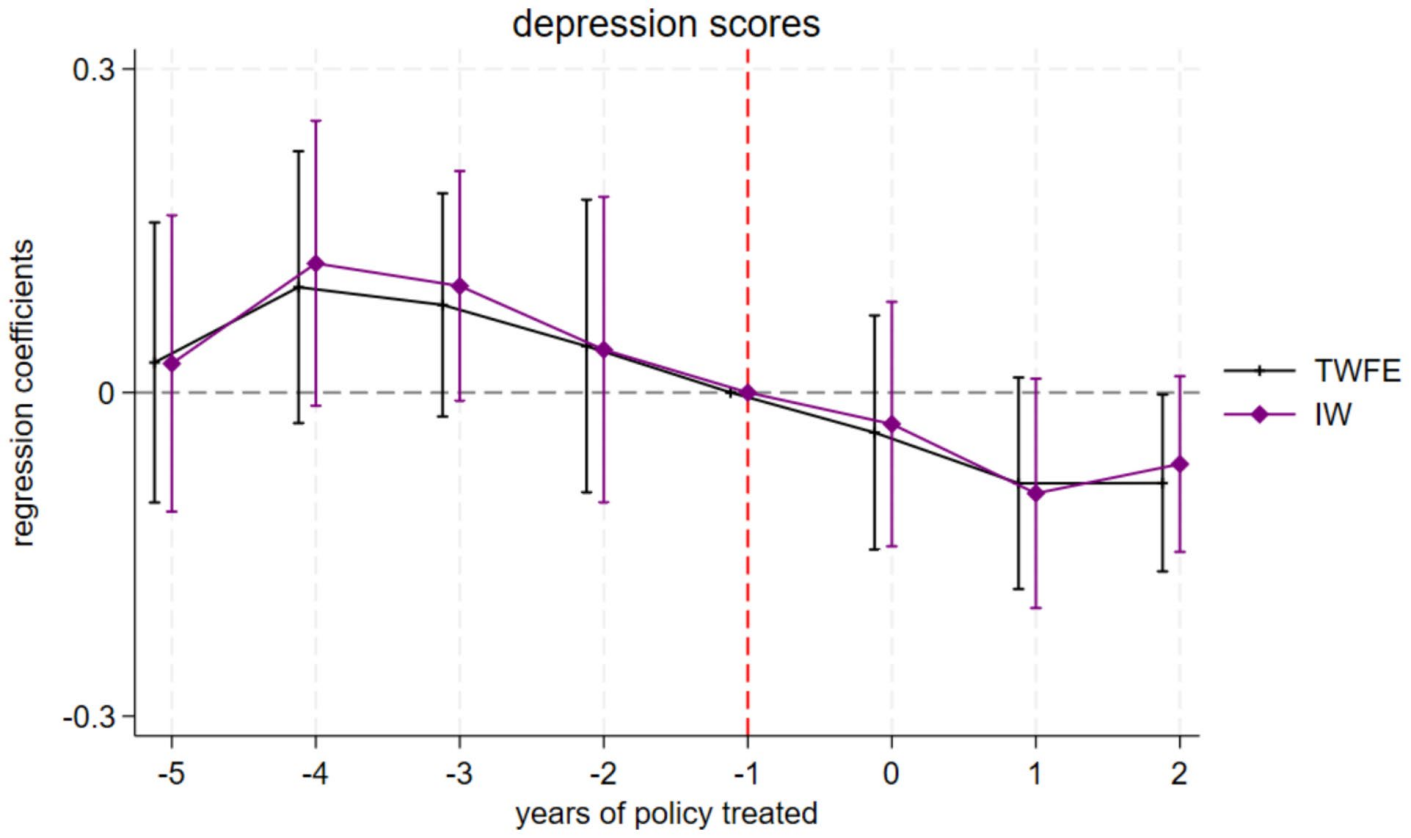
Parallel trends. TWFE, Two-way fixed effects model; IW, Interaction weights estimation method. The chart uses the previous phase (−1 phase) of the pilot policy as the base period, and the vertical solid line represents the 95% confidence interval.

The results of the parallel trend analysis were illustrated by the black line in Figure 2, which depicted the impact of the policy shock on the mental health status of older adults, as assessed through a two-way fixed effects model (TWFE). The analysis revealed that the coefficients for all indicators of depression scores prior to the implementation of the HCEC service reform pilot were not statistically significant. This indicates that there was no significant difference between the treatment group and the control group before the policy, and the parallel trend assumption set by the model was met. Furthermore, the policy effects of the HCEC exhibited distinct dynamic evolution characteristics in both the short and long term. This study primarily examined the long-term mechanisms resulting from the intensity of the pilot policy. In the medium to long term (after two periods), the HCEC service reform pilot demonstrated a sustained positive effect on enhancing the mental health levels of older adults.

The HCEC service reform pilot is being implemented gradually in five phases, embodying a progressive DID research framework. This approach necessitates adherence to the homogeneity assumption regarding treatment effects. However, given that the progressive DID method may have the problem of cross-period contamination, samples treated earlier may become the “bad control group” for samples treated later, thereby posing a threat to the validity of the parallel trend test. Consequently, the asymptotic DID method under the two-way fixed effects (TWFE) setting may have a problem of estimation bias. To address the potential bias arising from heterogeneous policy effects, this study employed the interactive weight estimation method (IW) as proposed by Sun et al. (13) to conduct a parallel trend test, thereby mitigating concerns associated with heterogeneous treatment effects. Utilizing the never-treated samples as the control group, the IW estimation results were illustrated as the purple line in Figure 2. The findings indicate that the depression scores passed the parallel trend test, and the effects of the pilot policy aligned with the results obtained from the TWFE analysis. Furthermore, for the event study results derived from the IW estimation, which excludes the “bad control group,” the treatment effect trend still fulfilled the homogeneity assumption. In summary, despite the potential implications of heterogeneous treatment effects, the baseline regression conclusions of this study remain credible.

### 3.3 Robustness assessment

#### 3.3.1 Exclude the competitive hypotheses

In the context of the gradual popularization of social older adult care services, China has not only introduced HCEC service reform policies but has also implemented a series of additional policies aimed at enhancing older adult care services during the same period. These initiatives may have implications for the mental health of older adults. Consequently, to demonstrate that the enhancement of older adults’ mental health was positively influenced by the HCEC service reform pilot, this section incorporated binary treatment variables representing three potentially competitive policies as control variables in the empirical regression analysis. This approach aims to mitigate the confounding effects of other policies.

The initial focus is on the long-term care insurance (LCI) policy. In 2016, the Ministry of Human Resources and Social Security of China issued the Guiding Opinions on Implementing the Pilot Program for the Long-Term Care Insurance System (14). This initiative aims to address the financial requirements associated with basic life care and medical services that are closely related to the fundamental needs of severely disabled individuals. The policy emphasized the health status of disabled persons, which may lead to confusion regarding the impact of the HCEC in relation to the long-term care insurance policy.

The second aspect pertained to the national medical-nursing integration pilot (NMNIP) policy. In 2016, the National Health and Family Planning Commission, in conjunction with the Ministry of Civil Affairs, issued a notice regarding the selection of national medical-nursing integration pilot units. This initiative encompassed 90 cities and districts nationwide, with a focus on the coordination and integration of medical service resources and older adult care service resources within the designated pilot areas. The NMNIP may also affect the research conclusions presented in this study.

The third initiative pertained to the pilot demonstration policy for smart health older adult care applications (SHEC). To facilitate the implementation of the Smart Health Elderly Care Industry Development Action Plan (2017–2020), the Ministry of Industry and Information Technology, the Ministry of Civil Affairs, and the National Health and Family Planning Commission of China issued a notice in 2017 regarding the execution of the pilot demonstration for smart health older adult care applications (15). Given that the pilot demonstration policy for smart health older adult care applications significantly influenced both the older adult care model and the physical and mental well-being of older adults, this study adopted the methodology of Jin and Yuqi (16) to construct a binary control variable for regression analysis, based on the designated demonstration base areas listed in the smart health older adult care application pilot. Table 4 presents the empirical results pertaining to the competitive hypotheses of the three aforementioned policies, which demonstrate the robustness of the main conclusions.

**Table 4:** The regression results with competitive hypotheses exclusion.

Variables	Controlled LCI policies (1)	Controlled NMNIP policies (2)	Controlled SHEC policies (3)
*DID*	−0.070**	−0.111***	−0.088***
	(0.034)	(0.038)	(0.033)
*R*-squared	0.042	0.038	0.038
Sample size (number)	34,375	34,375	34,375

(1) ** indicate statistically significant results: *p* < 0.05; *** indicate statistically significant results: *p* < 0.01. (2) DID: difference-in-differences. (3) This paper does not construct binary policy variables based on demonstration streets (towns) and demonstration enterprises. The reason is that the role of demonstration enterprises is to provide mature smart health care products and system platforms, focusing on the overall solution of smart health care, while the radiation range of demonstration streets (towns) is jurisdictional, and does not have a direct impact on other streets (towns) residents in the region.

#### 3.3.2 Additional robustness assessments

To account for the potential influence of time-invariant individual characteristics (e.g., gender, ethnicity), this study conducted a robustness test by including only individual fixed effects for the older adults. As shown in Column (1) of Table 5, HCEC still significantly improved the mental health of the older adults.

**Table 5:** Analysis of other robust regression.

Variables	Fixed effects (individual level) (1)	Control for higher-order trends of city characteristics (2)	Extended window period (3)	Municipality samples excluded (4)
*DID*	−0.071*	−0.083**	−0.077**	−0.115***
	(0.039)	(0.034)	(0.039)	(0.038)
*R*-squared	0.317	0.044	0.039	0.039
Sample size	33,799	34,375	21,796	30,089

(1) * indicates statistically significant results at *p* < 0.1; ** indicates statistically significant results at *p* < 0.05; *** indicates statistically significant results at *p* < 0.01. (2) The controlled variables are the same as in Table 3.

We also conducted an analysis by controlling for higher-order time trends of pre-policy city characteristics. To address the non-random selection of pilot cities, our baseline specification controls for a linear time trend interacted with pre-policy city characteristics. However, the effects of pre-policy city characteristics may be nonlinear. We therefore further introduced interaction terms between pre-policy city characteristics and quadratic and cubic time trends to account for such nonlinear patterns. The results are reported in Column (2) of Table 5, which show that the estimated impact of HCEC remained robust after controlling for these higher-order time trends.

This study extended the policy window period. Given that the China Family Panel Studies (CFPS) data contains individual-level tracking information, it facilitated the effective identification of whether individuals were consistently tracked across the surveyed years. The tracked data was used to assess the impact of the HCEC. Consequently, this research further extended the window period for the HCEC shock and utilized samples with a minimum of five surveyed periods to conduct baseline regression analysis. As demonstrated in Column (3) of Table 5, the regression results within the framework of the extended policy window period remained robust.

This study excluded special samples from municipalities directly governed by the Central Government. Given the disparities in regional development, the economic, cultural, and educational levels of these municipalities were significantly higher than those of other districts and counties, rendering them less comparable to the other pilot cities. Consequently, for the purpose of robustness analysis, this research omitted samples from municipalities directly under the Central Government. Column (4) of Table 5 presents the regression results following the exclusion of these samples. The findings indicated that, within the analytical framework that excluded samples from municipalities directly governed by the Central Government, the regression results remained robust.

### 3.4 Mechanism analysis

#### 3.4.1 The enhancement of physical health

**Figure 3:**
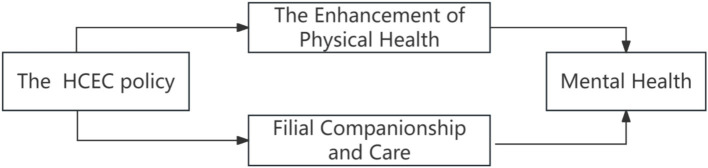
Mechanism analysis.

Physical health is a significant determinant of mental health (17). This study employed several dependent variables, including older adults’ self-rated health status, the incidence of illness within the past six months, the severity of illnesses, and hospitalization rates over the past year, to assess the impact of the HCEC policy on the health of older adults. Column (1) of Table 5 illustrates the effects of the HCEC policy implementation on the self-rated health scores of older adults. The findings indicated that the reform of HCEC substantially enhanced older adults’ self-assessment of their health, thereby improving their self-perception of health. It is important to note that this subjective measure of physical health is influenced not only by the HCEC policy but also by the increasing health-related needs of the older adults. Columns (2) to (4) of Table 6 present the effects of the HCEC on older adults’ health from an objective perspective. The results demonstrated that this reform not only mitigated the severity of illnesses among older adults but also decreased the likelihood of illness and hospitalization, thereby substantiating the “health” benefits associated with the HCEC. These findings are consistent with those of Wenxiu et al. (18). These results suggested that the integrated home- and community-based older adult care model offered older adults essential services, including daily care, medical assistance, and emotional support. This effectively enhanced their physical health and, consequently, their mental health, thereby embodying the concept of healthy aging (see Figure 3).

**Table 6:** Mechanism test of physical health enhancement.

Variables	Self-rated health status	Illness or not	Disease severity	Hospitalization or not
	(1)	(2)	(3)	(4)
*DID*	0.028	−0.026**	−0.059**	−0.019**
	(0.025)	(0.013)	(0.030)	(0.009)
*R*-squared	0.175	0.061	0.068	0.038
Sample size	40,388	34,810	35,076	34,811

(1) ** indicates statistically significant results: *p* < 0.05; (2) the controlled variables are the same as in Table 3.

#### 3.4.2 Filial companionship and care

Children’s companionship, care, and communication are critical factors in enhancing the mental health of older adults (19). The traditional family model of older adult care often prioritizes the provision of material needs, frequently neglecting the deeper spiritual and companionship needs of older individuals. Consequently, this practice of prioritizing material over spiritual needs leads to the marginalization of older adults within the family structure, ultimately adversely affecting their mental health (20). Table 7 presents the findings related to the filial companionship and care mechanisms resulting from the reform of HCEC. Column (1) illustrates the impact of the HCEC on the frequency of visits between children and their older relatives. The results indicate that the implementation of the HCEC significantly increased the frequency of visits between older adults and their children. This suggests that during the HCEC’s implementation period, local governments effectively promoted the new paradigm of HCEC, raising awareness of the importance of ensuring a fulfilling life for older adults. Furthermore, this initiative encouraged families to actively engage in community-organized home care training, thereby increasing the level of care and companionship provided by children to their older relatives. Column (2) examines the impact of the HCEC on the frequency of communication between children and older adults, revealing that the HCEC significantly increased intergenerational communication. These findings indicate that the HCEC substantially enhanced children’s willingness to care for older adults within the family, thereby fostering increased caregiving behaviors and ultimately improving the mental health of older adults (see Figure 3).

**Table 7:** Mechanism test of filial companionship and care.

Variables	The frequency of meetings between children and the older adults	The frequency of communication between children and the older adults
	(1)	(2)
*DID*	0.501*** (0.162)	0.337** (0.156)
*R*-squared	0.184	0.196
Sample size	24,363	24,363

(1) ** indicates statistically significant results: *p* < 0.05; *** indicates statistically significant results: *p* < 0.01; (2) the controlled variables are the same as in Table 3.

### 3.5 Heterogeneity effects

Due to the disparities in mental health among the older adults, the extent of the impact of the HCEC also varied. This part focused on critical factors, including gender, educational attainment, and self-reported health status, to conduct a heterogeneity analysis.

#### 3.5.1 Gender heterogeneity

The examination of gender differences has been a central focus within demographic theory research (21). Evidence suggests that older women are more vulnerable to physical illness, psychological disorders, cognitive impairments, and various other issues when compared to their male counterparts (22). Consequently, this study conducted a group regression analysis based on the initial regression findings to examine gender differences. The results are presented in Panel A of Figure 3. The HCEC has been shown to significantly enhance the mental health of both older men and women; however, the positive impact was notably more prominent among women. This disparity may be attributed to the HCEC’s role in amplifying women’s demand for healthcare within home- and community-based older adult care settings. Furthermore, it encouraged older women to enhance their self-care capabilities and quality of life, thereby effectively reducing the mental health disparity between genders. This outcome was also in line with the expected social benefits of the HCEC.

#### 3.5.2 Educational level

**Figure 4:**
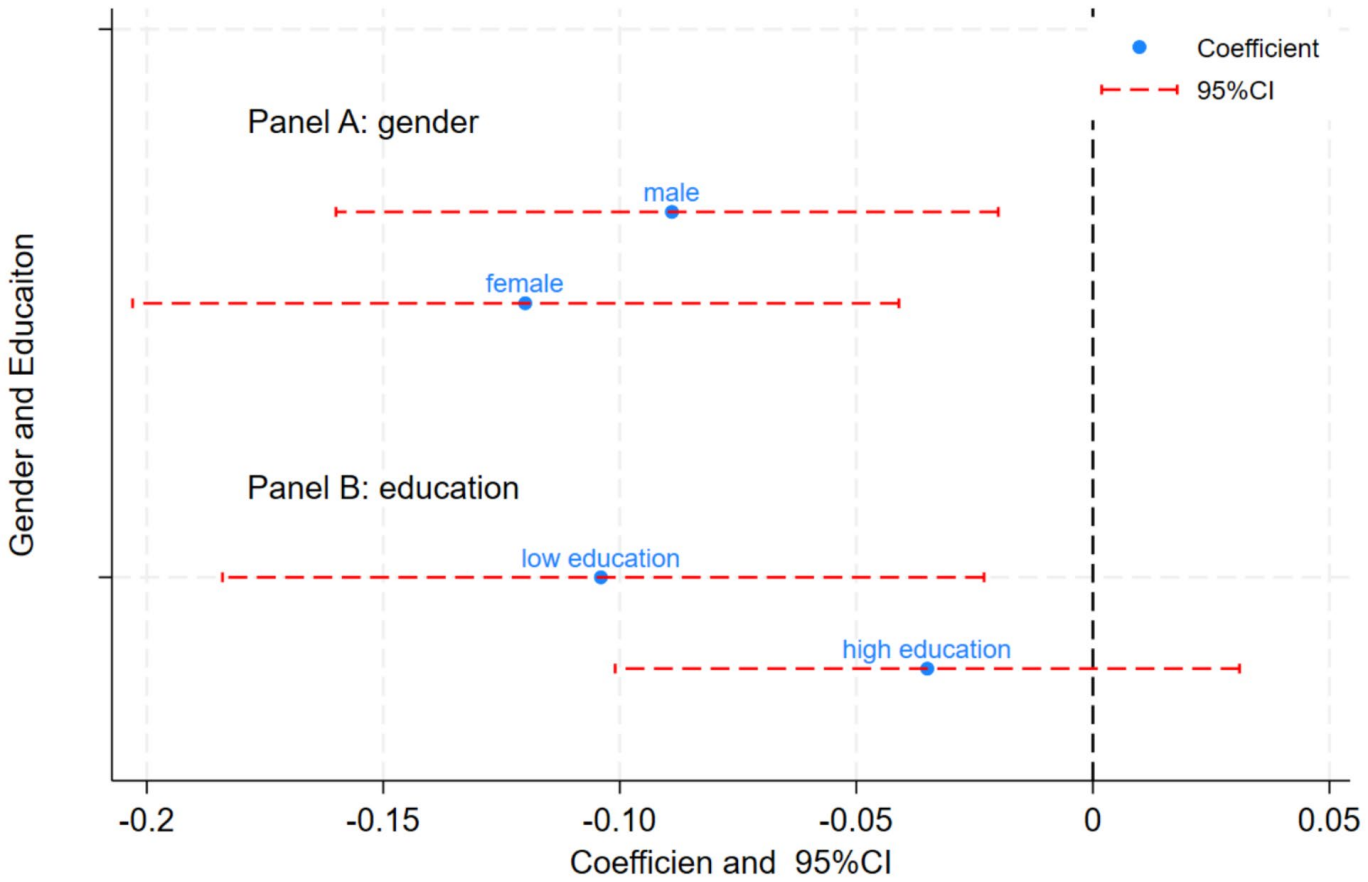
Heterogeneity analysis of gender and educational level. **(A)** Gender heterogeneity analysis. **(B)** Educational level heterogeneity analysis.

It is hypothesized that older adults with higher levels of education tend to exhibit greater receptiveness to new things, which subsequently influences their access to essential resources such as basic necessities and medical care. This access is crucial for improving their health outcomes and modifying detrimental lifestyle habits (23). Consequently, this study employed education levels for the purpose of group analysis. Specifically, individuals with education levels below junior high school were categorized as the low-education group, while those with junior high school education or higher were classified as the high-education group. The analysis of educational level heterogeneity is presented in Panel B of Figure 4. The HCEC demonstrated a significant positive effect on the depression scores of older adults with low education levels, whereas no significant impact was observed for those with high education levels. In order to determine whether the policy’s impact across different educational levels reflects a true ‘structural difference’ rather than simply a difference in estimation precision. To ensure both proper identification of fixed effects and valid application of clustered standard errors, we performed the Chow test across the two education groups of older individuals. The interaction term between education group and the HCEC policy yielded a coefficient of −0.202 and was statistically significant at the 1% level (*p* < 0.01), as reported in Column 5 of Table A1. These results confirm that HCEC has a structurally heterogeneous effect on older people with different educational attainment. According to the seventh national population census conducted in 2020, among the population aged 60 and above in China, 36.69 million individuals possessed a high school education or higher, namely, the majority of older adults remain in the low-education group. These empirical findings suggest that the HCEC plays a constructive role in promoting mental health levels and enhancing quality of life, thereby underscoring the positive impact of the HCEC on the mental health of older adults with low education levels.

#### 3.5.3 Chronic diseases and disability status

**Figure 5:**
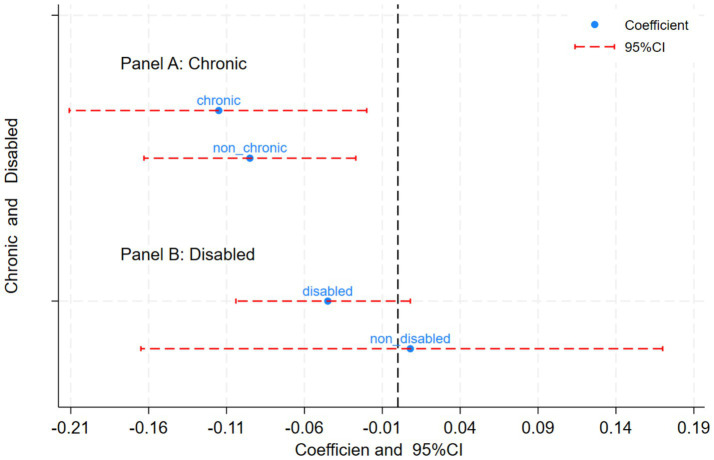
Heterogeneity analysis of chronic diseases and disability. **(A)** Chronic diseases heterogeneity analysis. **(B)** Disability heterogeneity analysis. Based on CFPS data, this paper aggregates seven indicators of disability. If an individual has one of these disabilities, they are defined as disabled; otherwise, they are defined as non-disabled. The seven disability indicators are: ability to engage in outdoor activities independently, ability to eat independently, ability to perform kitchen activities independently, ability to use public transportation independently, ability to shop independently, ability to perform personal hygiene independently, and ability to do laundry independently.

Older adults are the primary vulnerable group affected by chronic diseases and disabilities [In this study, nearly 80% of the participants had no chronic diseases, and nearly 50% were disabled individuals.]. Research indicates that older adults with chronic diseases have a higher risk of depression (24), and disability also significantly influences mental health levels (25, 26). Therefore, the present study specifically examined the groups with chronic diseases and disabilities to assess the impact of the HCEC. Panel A of Figure 5 shows the results of the HCEC’s impact on the chronic disease and non-chronic disease groups. The findings showed that the HCEC has significantly improved the mental health of older adults without chronic diseases. According to CFPS data, more than 80% of the participants do not have chronic diseases; thus, our findings underscored the significant role of accessibility in the policy implemented among older adults, which was in line with social needs. Panel B shows the policy’s impact on the disabled and non-disabled groups; the older adult care policy effectively improved the mental health of the disabled group. This result indicated that the policy has a welfare-oriented attribute, leading to improvements in mental health for both disabled individuals and those without chronic diseases.

## 4 Discussion

The reform of HCEC holds significant importance in addressing the national strategy for healthy aging and fostering the high-quality development of older adult care services. The emergence of HCEC in China commenced relatively late and remains in the initial stage of development at present. The mechanism analysis in this study indicated that the physical health of older adults positively influences the enhancement or maintenance of their mental health. Notably, substantial improvements in the mental health of older adults can be achieved through the enhancement of physical health status and the promotion of health management awareness (27, 28). Furthermore, the provision of emotional support by children emerged as a critical factor influencing the mental health of older adults, which was consistent with the findings from other scholars (29). Home- and community-based older adult care policies provide professional daily care, medical care, cultural and sports activities, and other instrumental support and public companionship resources for older adults through government public service provision, thereby compensating for the functional gaps in family older adult care. On the one hand, community older adult care services replace part of the daily care work previously undertaken by adult children, reducing their time and energy costs in older adult care and creating favorable conditions for children to engage in affective companionship such as visits and communication. On the other hand, community older adult care platforms established by relevant policies provide scenario-based carriers for intergenerational interaction (e.g., community-organized filial piety activities, parent–child classes for older adults), extending intergenerational companionship from the “private family domain” to the “public community domain” and stimulating the provision of affective and companionship support from adult children (30). From the perspective of supply–demand matching in social support, policies alleviate the supply–demand imbalance in family older adult care by precisely meeting the basic care needs of older adults, changing children’s older adult care behavior from “passive material coping” to “active emotional investment.” This constitutes one of the theoretical mechanisms through which such policies significantly increase the frequency of intergenerational visits and communication.

Social Support Theory classifies social support into four dimensions (instrumental, emotional, informational, and companionship support) and argues that the integrity of an individual’s social support network affects their behavioral choices and psychological status. As the core support system, the family relies on external environmental conditions to function effectively (31, 32). Under China’s traditional family-based older adult care model, adult children are the main providers of social support for older adults. However, population aging, low fertility rates, and large-scale internal migration have weakened the instrumental capacity of family care. Constrained by limited time and energy, children can hardly balance material care and emotional companionship for their older parents, resulting in an older adult care dilemma marked by adequate material support but insufficient emotional companionship (33, 34). Community-based home care policies compensate for family care gaps by providing professional instrumental support and public companionship resources. Specifically, community care reduces the burden of children’s caregiving, enabling them to engage in emotional interactions, and the policy-built community platforms offer scenarios for intergenerational interaction, extending companionship from the private family atmosphere to the public community domain and stimulating children’s emotional and companionship support provision (34, 35). From the perspective of social support supply–demand matching, the policy alleviates family care imbalances, shifting children’s care behavior from passive material provision to active emotional engagement, which is one of the mechanisms explaining the policy’s significant positive impact on the frequency of intergenerational visits and communication.

Heterogeneity analysis revealed that the benefits of the HCEC were influenced by demographic characteristics, including gender, educational level, disability status, and the presence of chronic diseases among older adults. Consequently, it is imperative that targeted policies be developed to address the needs and protect vulnerable older individuals, such as those with low educational levels, disabilities, and females, thereby facilitating their access to services. These vulnerabilities, resulting from the prolonged accumulation of health risks, are not solely linked to personal physical health but are also closely related to the social environment and personal lifestyle (36, 37). Therefore, targeted prevention and intervention strategies are essential to assist individuals with chronic diseases in addressing their health problems.

For instance, for disabled older adults, priority should be given to their core demands for daily living and medical care. Service provision should be strengthened in community home care, professional rehabilitation, and home-based chronic disease management, so as to improve the care support network for disabled older (38). For those with low educational attainment, accessible and visualized communication strategies should be adopted to promote policy literacy and service access, thereby reducing barriers to policy understanding and service utilization. Given the greater mental health vulnerability and stronger emotional needs of older women, community-based older adult care systems should incorporate tailored services including emotional support, psychological counseling, and social participation to alleviate loneliness and depression. Such targeted interventions will effectively narrow disparities in older adult care services and mental health support across different groups.

The inclination of the HCEC to prioritize vulnerable older adults underscores the positive impact of older adult care service policies in promoting healthy aging. Simultaneously, during the policy implementation process, it is crucial to consider the demographic characteristics of vulnerable individuals. This can be achieved through cross-departmental coordination and collaboration, which can help mitigate policy fragmentation, avoid the “welfare cliff” phenomenon during the process of policy implementation (39), and ensure the fairness and effectiveness of policy benefits.

This study is the first comprehensive analysis of the individual welfare impacts associated with five distinct batches of HCEC pilot policies. The dataset encompasses 125 representative cities in China, establishing a causal relationship between HCEC and the mental health of older adults. Furthermore, it thoroughly investigates the medium- to long-term policy effects and underlying mechanisms. Additionally, this study controlled for the competitive influences of the long-term care insurance policy, the national medical-nursing integration pilot policy, and the smart health older adult care application pilot demonstration policy, thereby mitigating potential confounding effects. Consequently, the findings of this study are representative and reliable. Moreover, the results identify potential directions for future research. Specifically, the decline in cognitive function among older adults, which is closely linked to mental health, represents a significant area of social concern presently. Thus, investigating how these policies enhance cognitive function in older adults will be a pertinent direction in future research. Finally, the participants in this study are from the largest developing country, which provides valuable insights for the formulation of older adult care service policies in other developing countries.

## 5 Conclusion

This study utilized data from the HCEC reform pilot at the city (district) level, and the China Family Panel Studies, assessed the effectiveness of such reforms in enhancing the mental health of older adults within the context of an aging population. Notably, the reform demonstrated a more prominent impact on older women with low educational levels and disabilities.

## Data Availability

Publicly available datasets were analyzed in this study. This data can be found at: http://www.isss.pku.edu.cn/cfps/.

## Author’s note

Previous research on the effects of this policy has mainly focused on health status, quality of life, and other perspectives among older adults, while studies from a psychological perspective remain insufficient. To the best of our knowledge, this study is the first to comprehensively examine the impact of this policy on the mental health of older adults. We identify the causal relationship between home- and community-based care services and the mental health of older adults, conduct heterogeneity analysis, and further explore the long-term effects and underlying mechanisms of the policy. This study also controls for potential confounding policy effects to rule out interference from other related policies. Consequently, these findings are representative and reliable. The participants were drawn from 125 representative cities in the world’s largest developing country, which provides valuable insights for the formulation of older adult care policies and the implementation of healthy aging initiatives in other developing countries.

## Appendix

**Table A1:** Heterogeneity analysis of gender and educational level.

Variables	Gender		Education level		
	Male	Female	Junior school and below	Junior school and above	Chow test
	(1)	(2)	(3)	(4)	(5)
*DID*	−0.089**	−0.122***	−0.104**	−0.035	−0.031
	(0.036)	(0.041)	(0.041)	(0.034)	(0.037)
Edu group*DID					−0.202***
					(0.039)
R-squared	0.054	0.046	0.041	0.129	0.039
Sample size	17,495	16,880	25,389	8,986	34,375

(1) * indicates statistically significant results at *p* < 0.1; ** indicates statistically significant results at *p* < 0.05; *** indicates statistically significant results at *p* < 0.01. (2) The controlled variables are the same as in Table 1. (3) Edu group*DID denotes the interaction term between educational group and the policy.
